# Supplementary material for: Ethyne Functionalized Meso-Phenothiazinyl-Phenyl-Porphyrins: Synthesis and Optical Properties of Free Base Versus Protonated Species
Source: Molecules. 2020 Oct 4;25(19):4546. doi: 10.3390/molecules25194546 (PMC7583012; doi:10.3390/molecules25194546)
Supplement: Supplementary file 1 [file molecules-25-04546-s001.pdf]

Ethyne functionalized meso-phenothiazinyl-phenyl-porphyrins: synthesis and optical properties of free base versus protonated species.

Eva Molnar[1], Emese Gál[1]*, Luiza Gaina[1], Castelia Cristea[1]* and Luminita Silaghi-Dumitrescu[1]

Table of contents:

NMR spectra of compounds **2a, 3a, 2c, 3c, 4a**
HRMS spectra of compounds **3a, 2c, 3c, 5a**

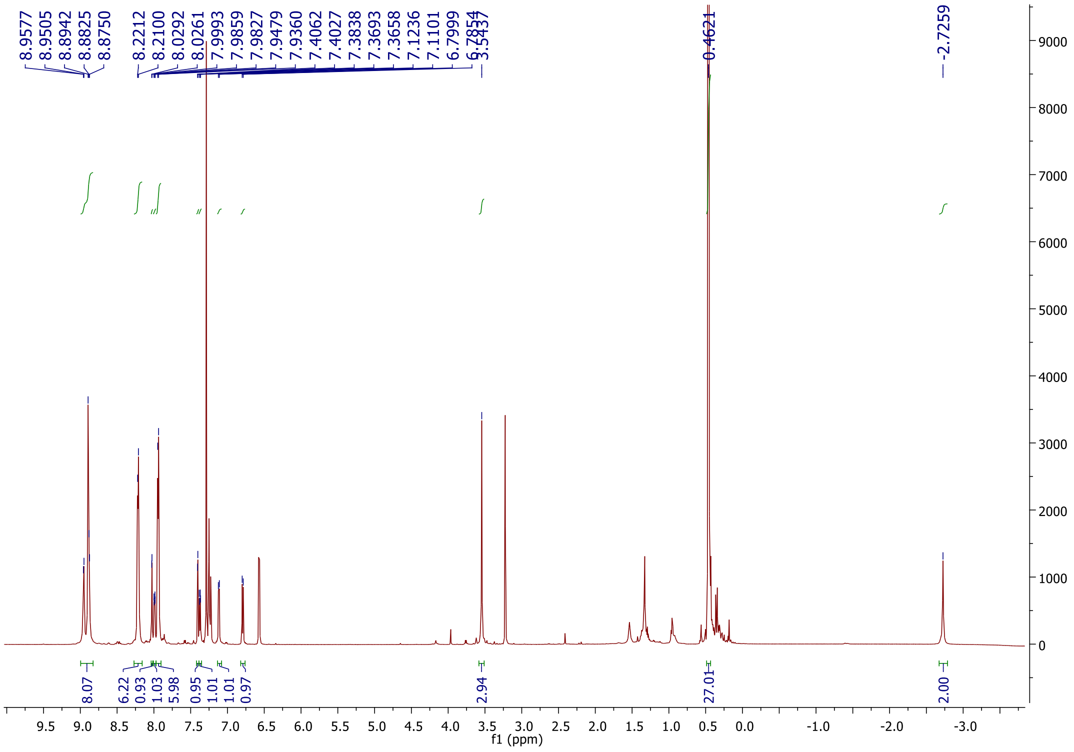

Figure S1. ^{1}H-NMR spectrum of compound **2a** ($CDCl_3$, 600 MHz)

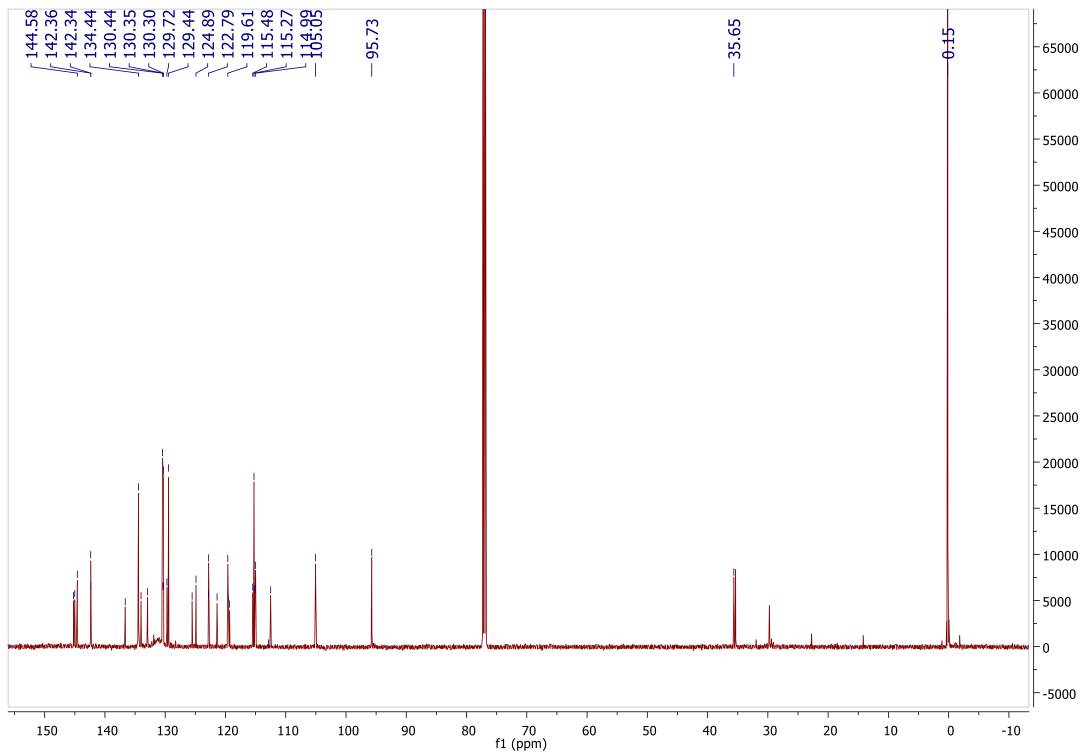

Figure S2. ^{13}C-NMR spectrum of compound **2a** ($CDCl_3$, 150 MHz)

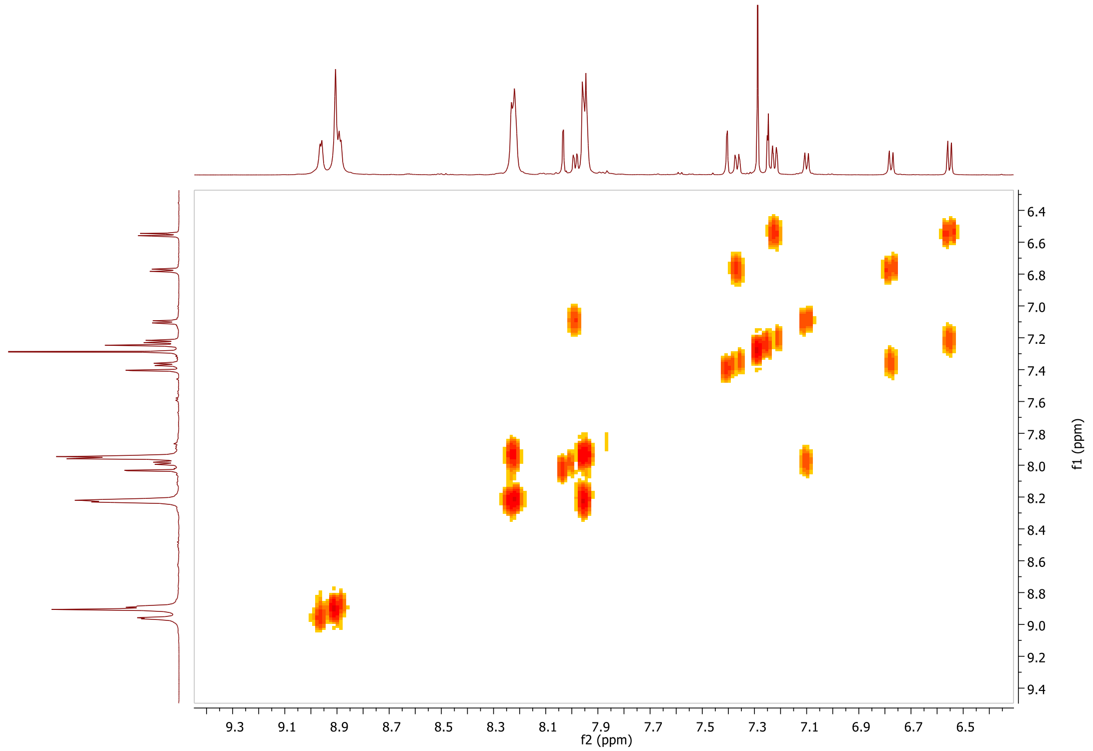

Figure S3. (^{1}H-^{1}H) COSY spectrum of compound **2a** (aromatic part, $CDCl_3$)

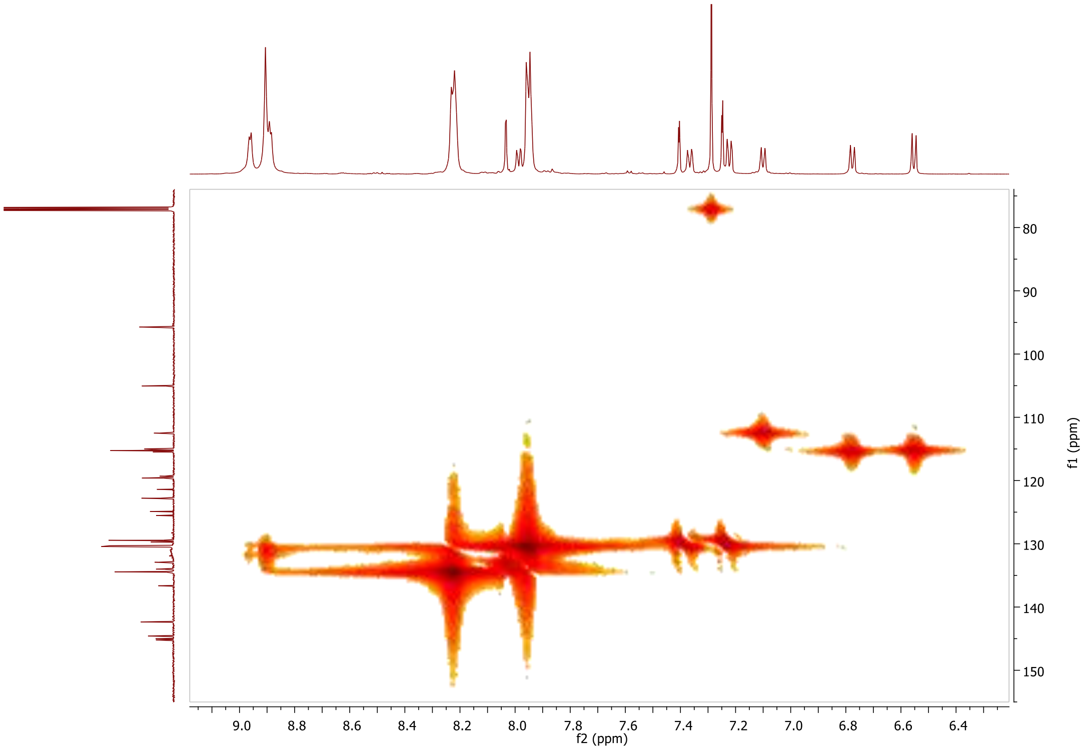

Figure S4. (^{1}H-^{13}C) HMQC spectrum of compound **2a** (aromatic part, $CDCl_3$)

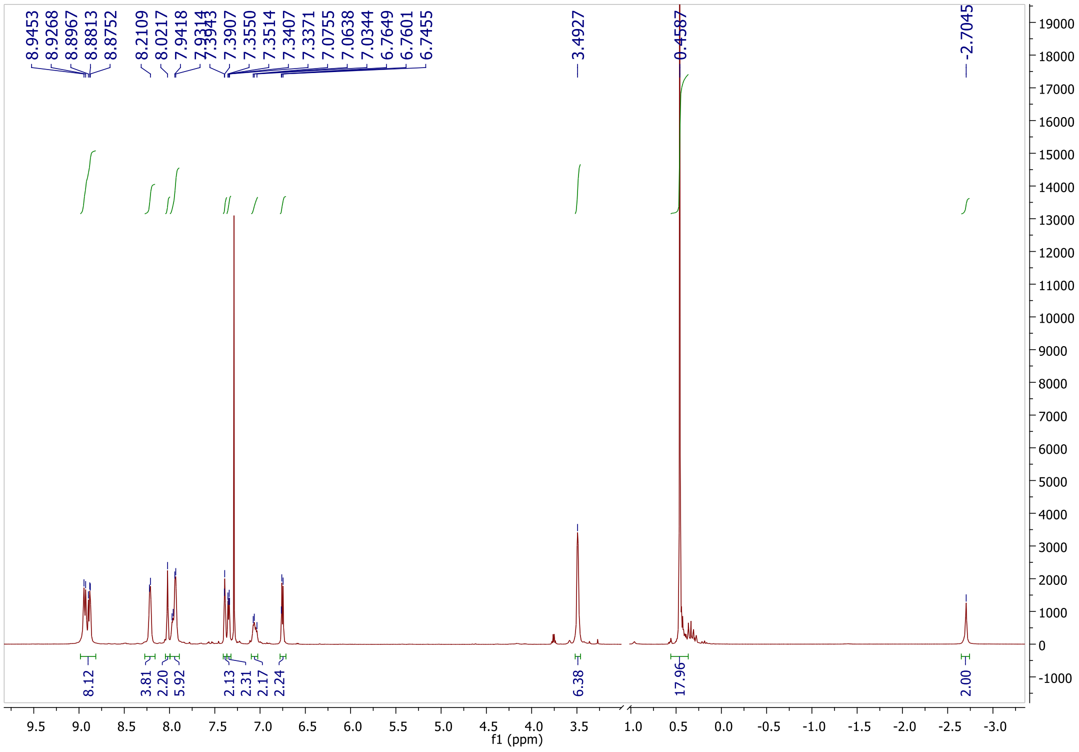

Figure S5. ^{1}H-NMR spectrum of compound **3a** ($CDCl_3$, 600 MHz)

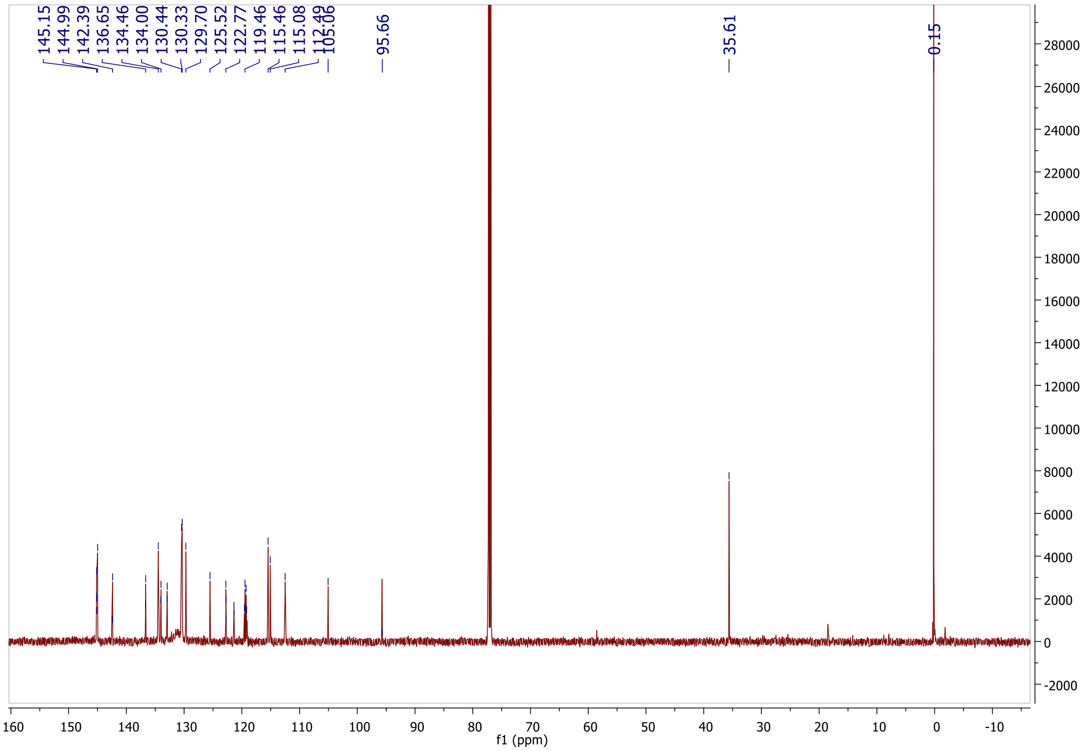

Figure S6. ^{13}C-NMR spectrum of compound **3a** ($CDCl_3$, 150 MHz)

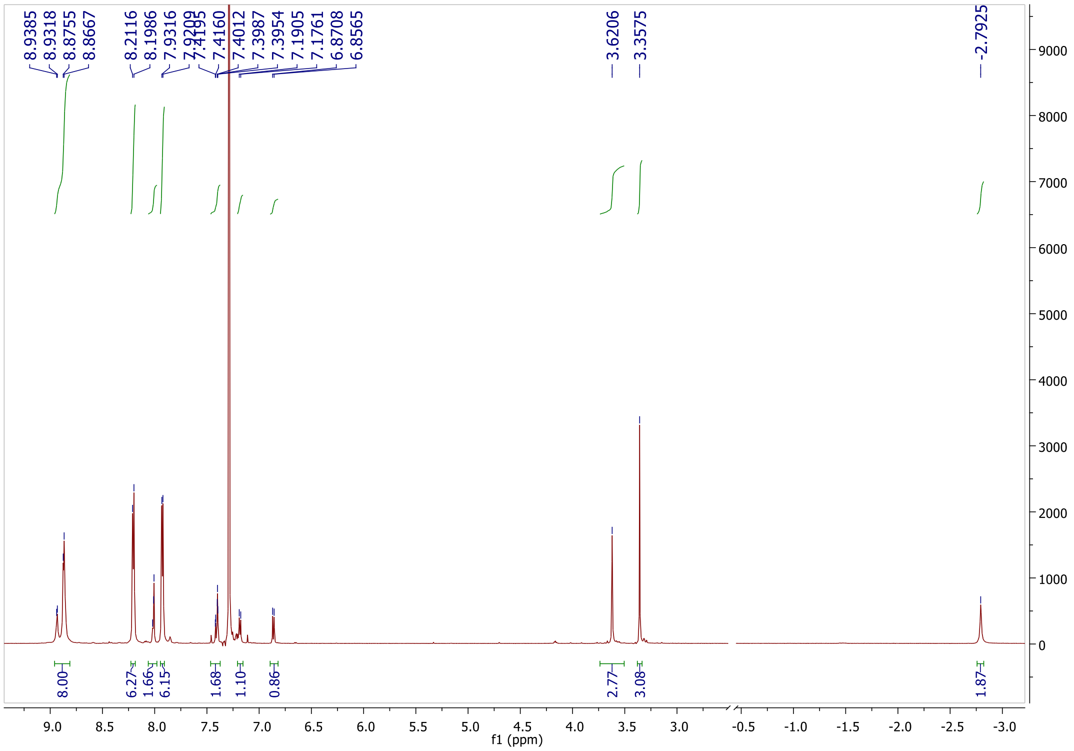

Figure S7. ^{1}H-NMR spectrum of compound **2c** ($CDCl_3$, 600 MHz)

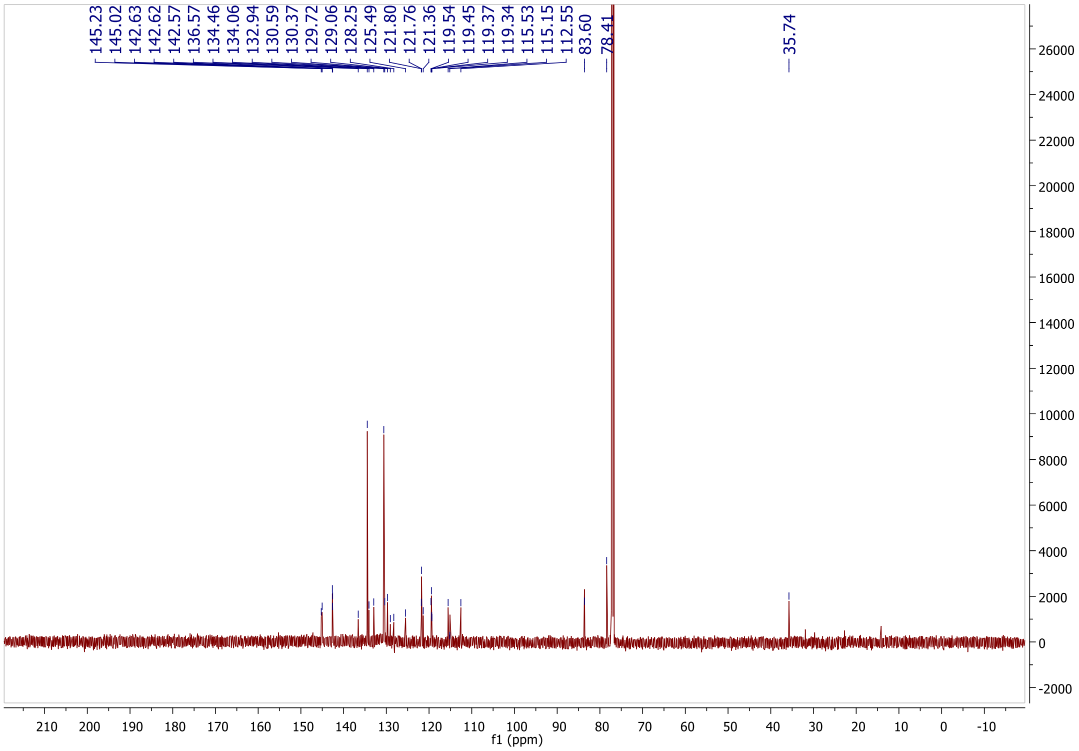

Figure S8. ^{13}C-NMR spectrum of compound **2c** ($CDCl_3$, 150 MHz)

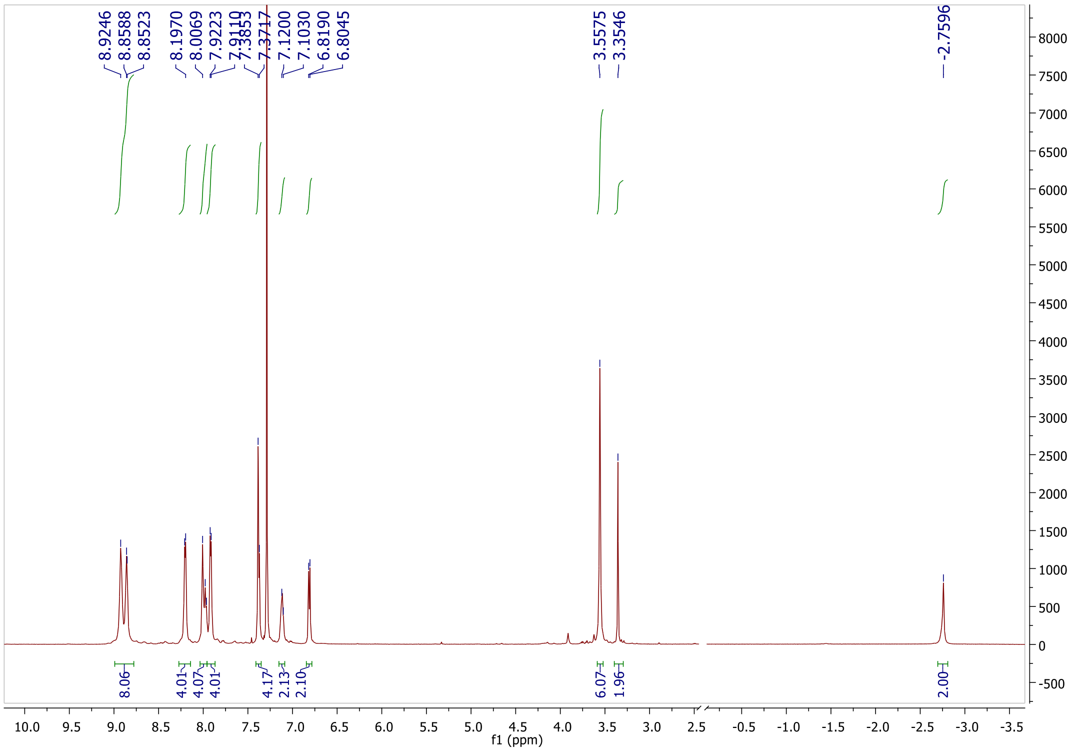

Figure S9. ^{1}H-NMR spectrum of compound **3c** ($CDCl_3$, 600 MHz)

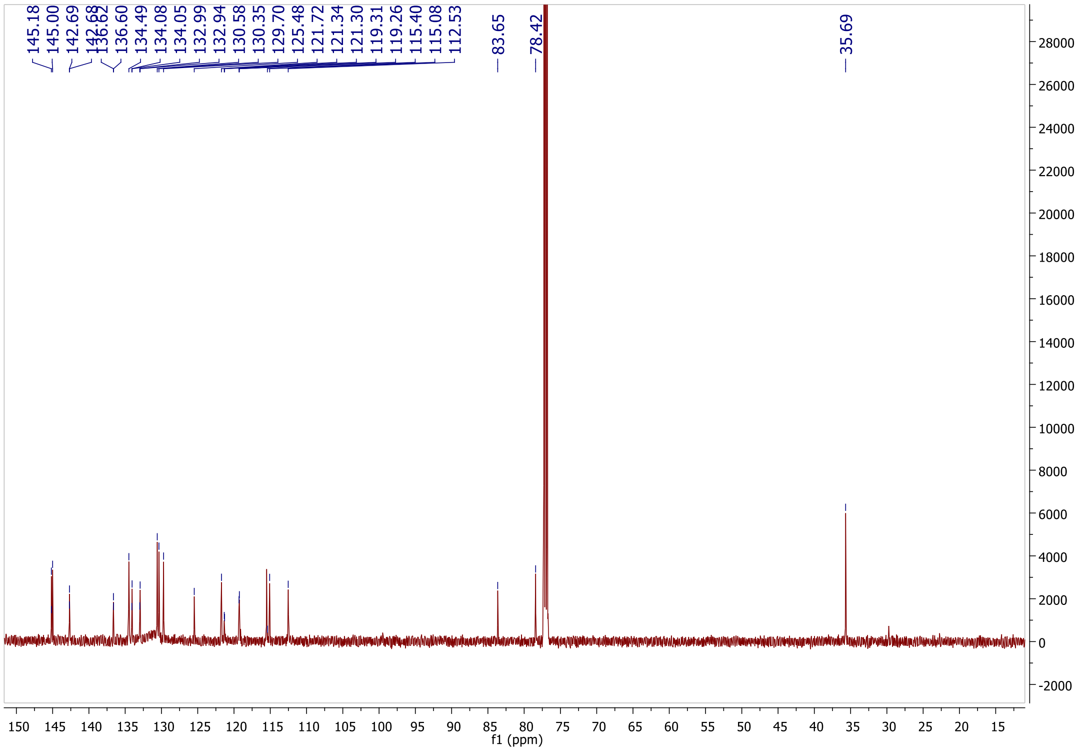

Figure S10. ^{13}C-NMR spectrum of compound **3c** ($CDCl_3$, 150 MHz)

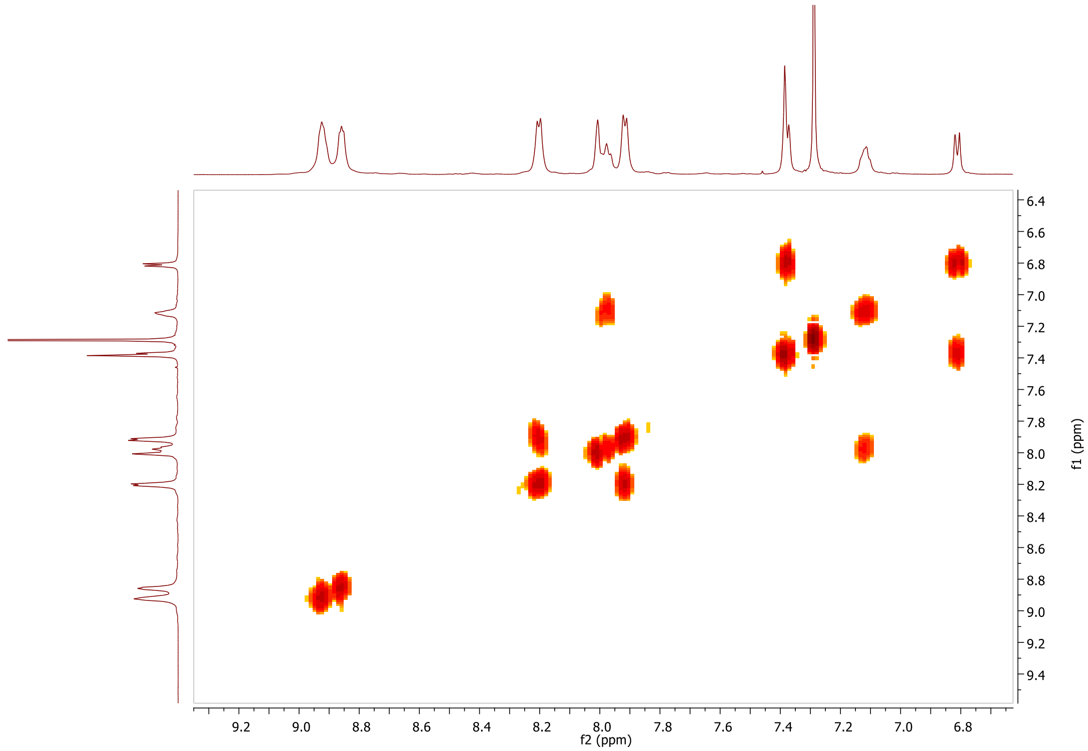

Figure S11. (^{1}H-^{1}H) COSY spectrum of compound **3c** (aromatic part, $CDCl_3$)

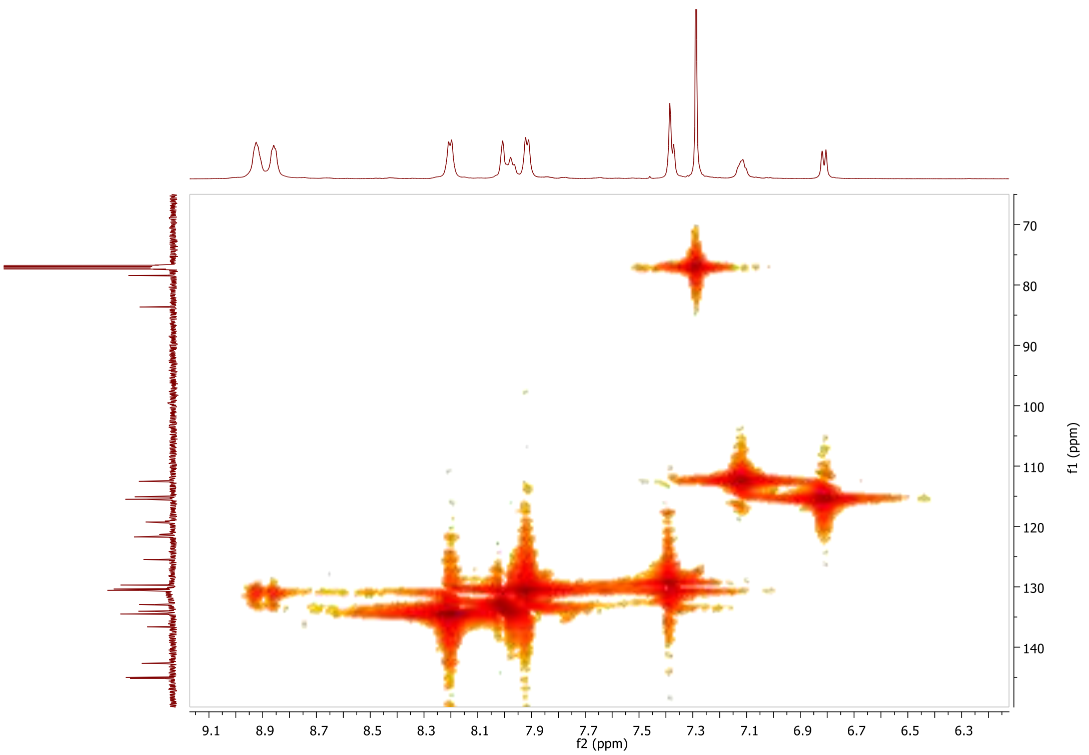

Figure S12. (^{1}H-^{13}C) HMQC spectrum of compound **3c** (aromatic part, $CDCl_3$)

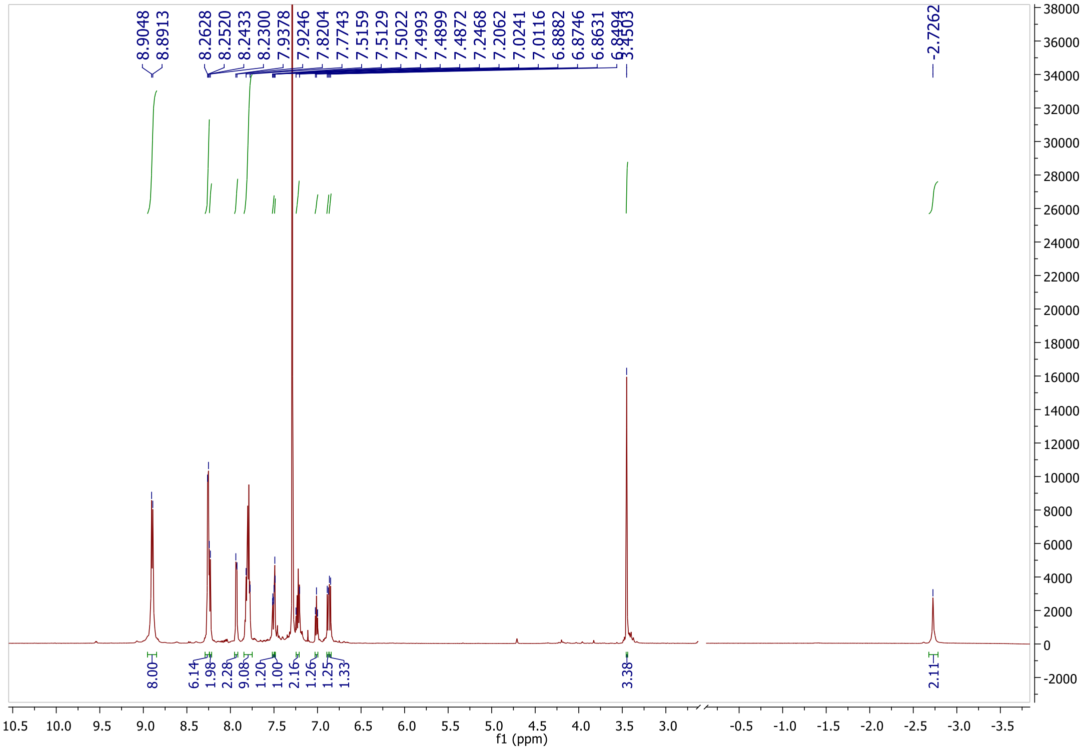

Figure S13. ^{1}H-NMR spectrum of compound **4a** ($CDCl_3$, 600 MHz)

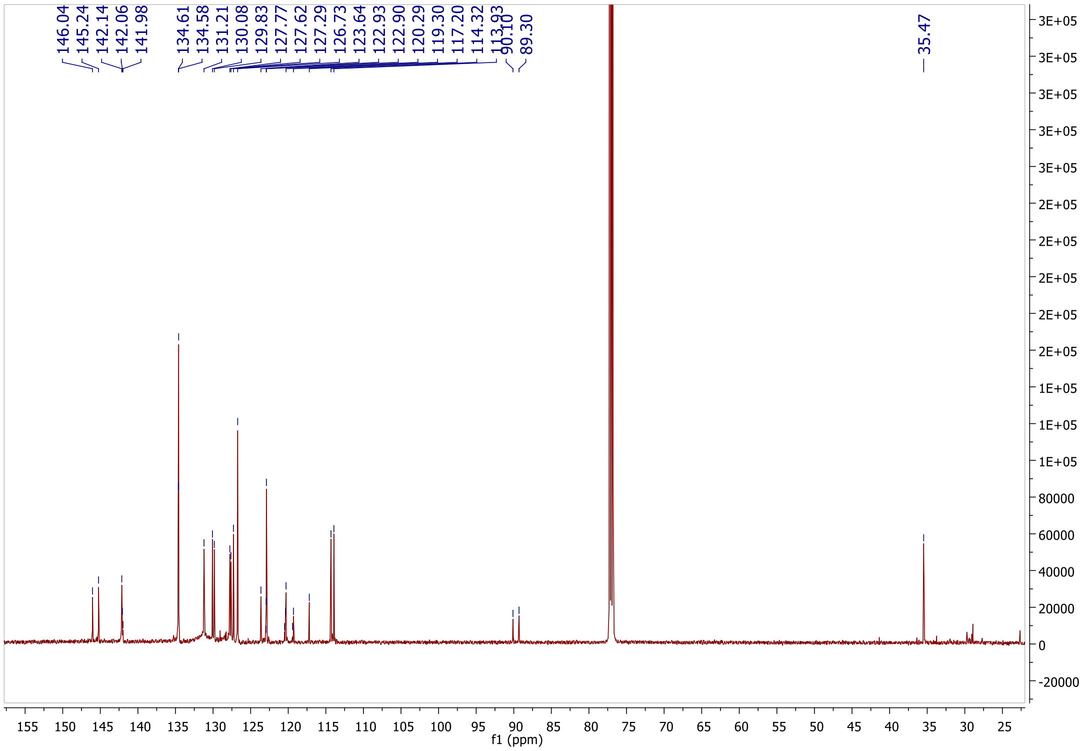

Figure S14. ^{13}C-NMR spectrum of compound **4a** ($CDCl_3$, 150 MHz)

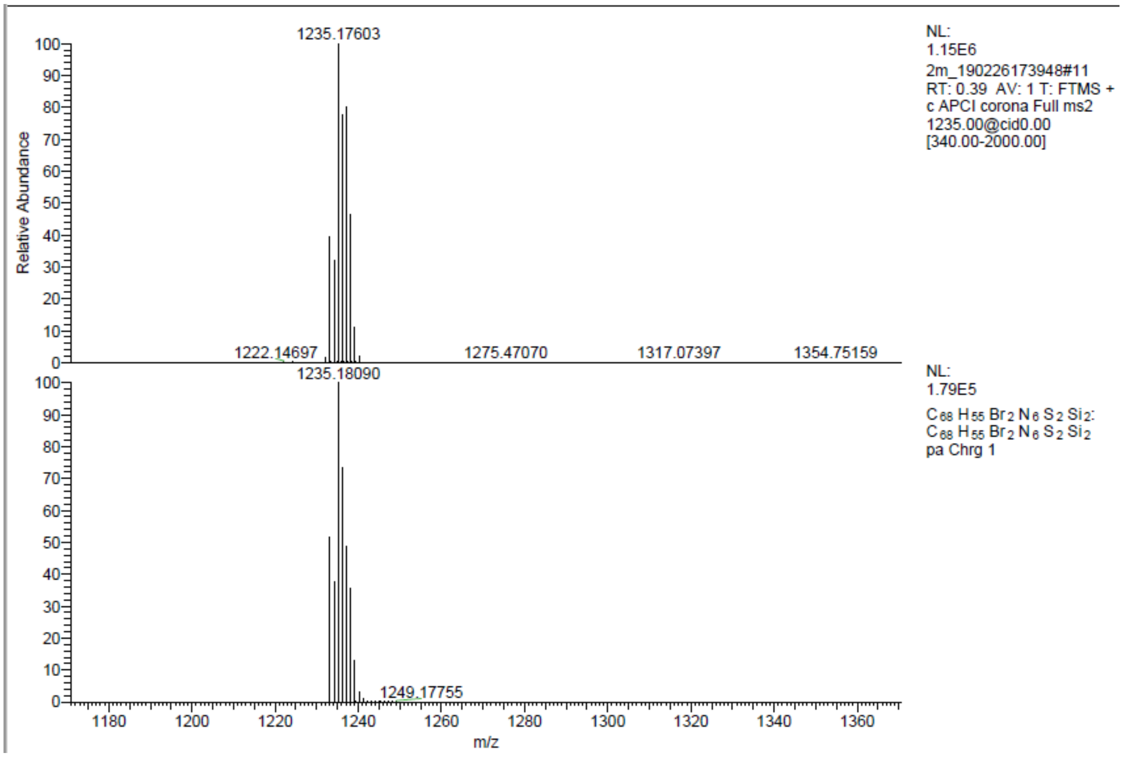

Figure S15. HRMS (APCI+) spectrum of compound **3a**

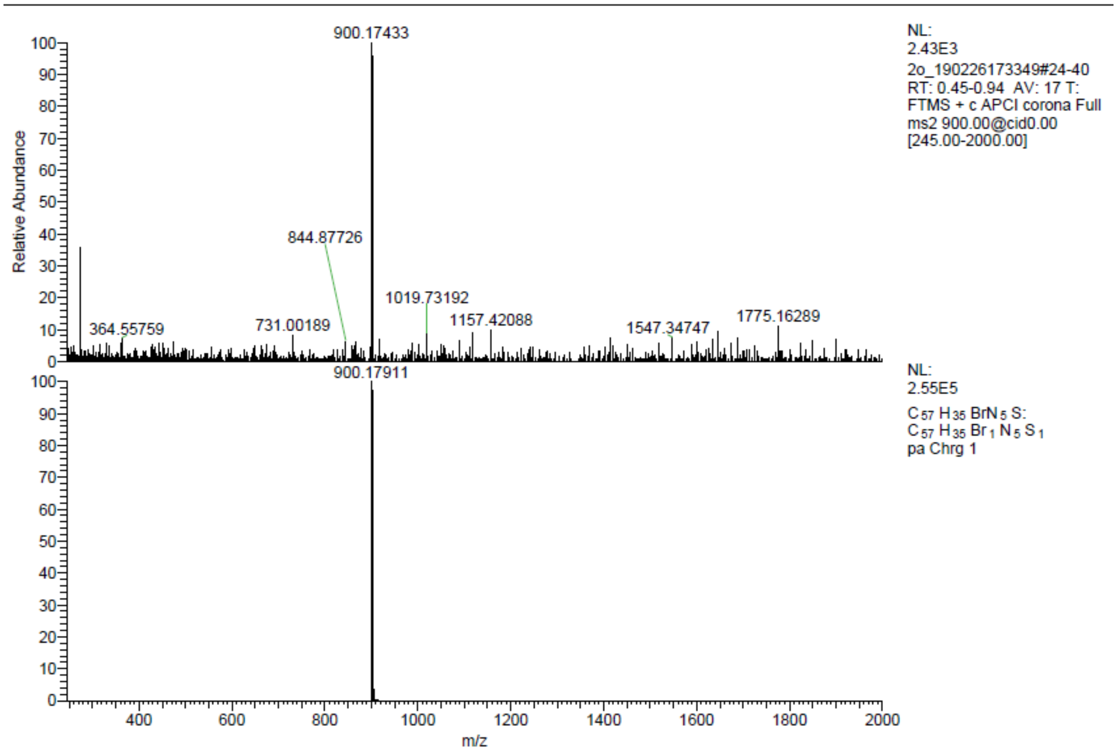

Figure S16. HRMS (APCI+) spectrum of compound **2c**

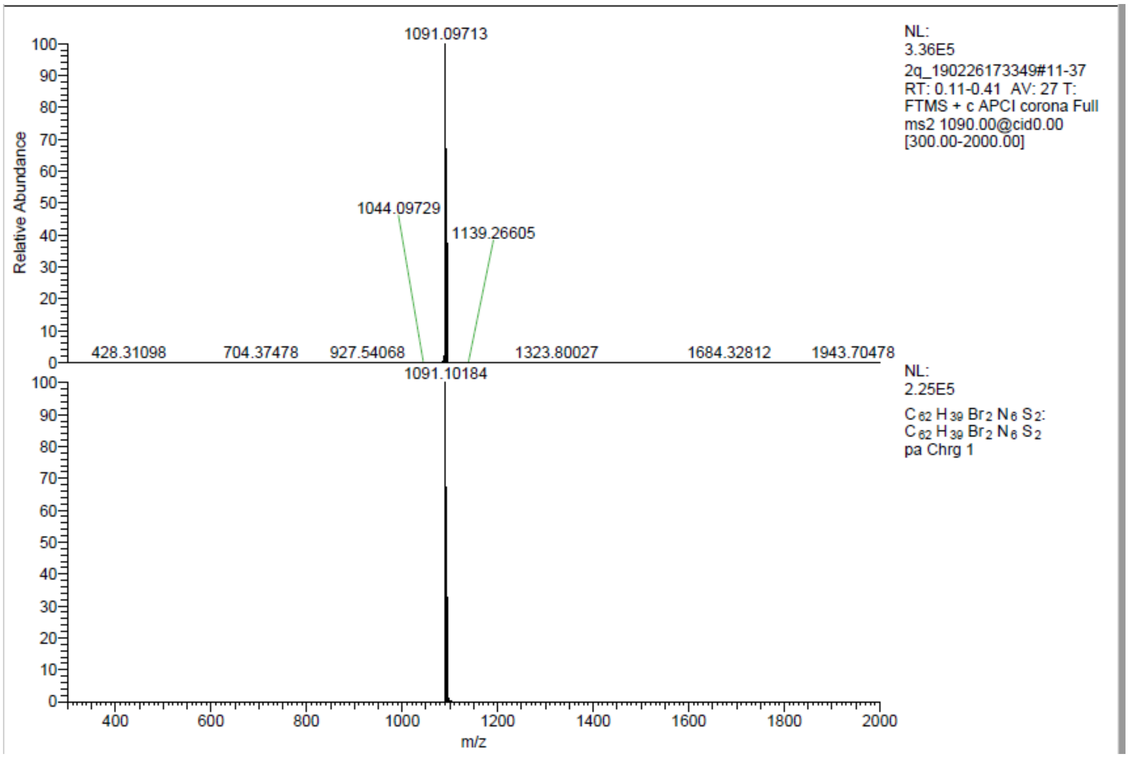

Figure S17. HRMS (APCI+) spectrum of compound **3c**

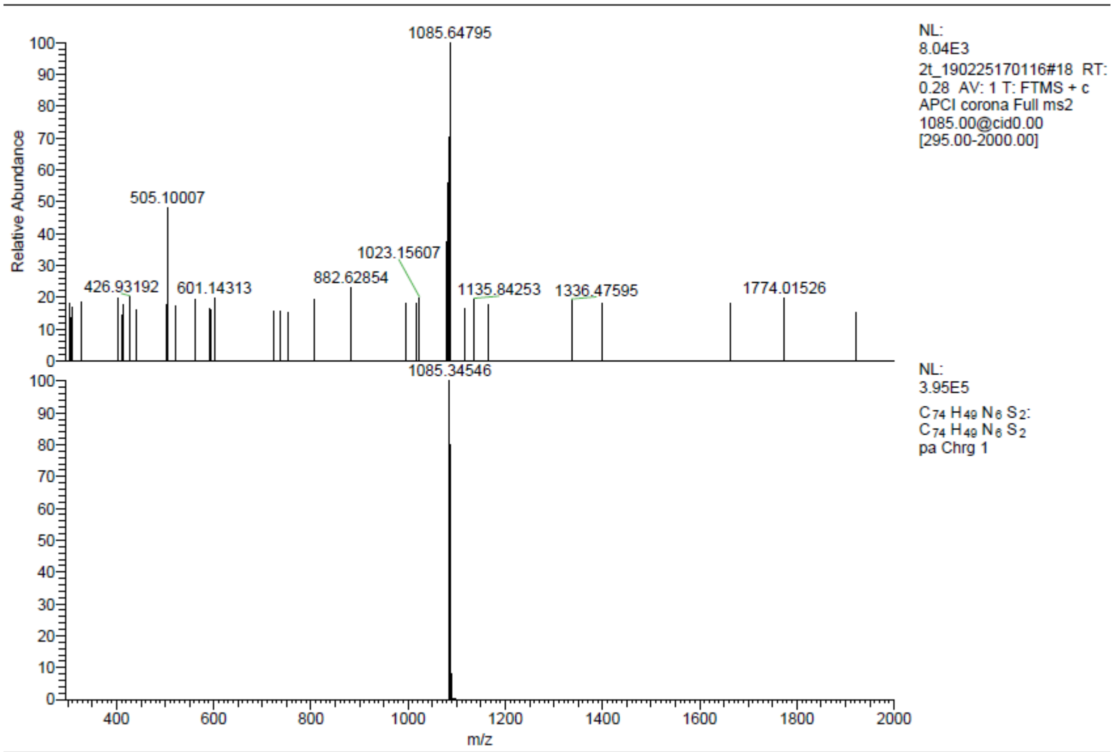

Figure S18. HRMS (APCI+) spectrum of compound **5a**
